# Mechanisms Underlying Influence of Bioelectricity in Development

**DOI:** 10.3389/fcell.2022.772230

**Published:** 2022-02-14

**Authors:** Laura Faith George, Emily Anne Bates

**Affiliations:** Department of Pediatrics, University of Colorado School of Medicine, Aurora, CO, United States

**Keywords:** bioelectricity, ion channels, signaling, signaling pathways, prolifieration, apoptosis

## Abstract

To execute the intricate process of development, cells coordinate across tissues and organs to determine where each cell divides and differentiates. This coordination requires complex communication between cells. Growing evidence suggests that bioelectrical signals controlled via ion channels contribute to cell communication during development. Ion channels collectively regulate the transmembrane potential of cells, and their function plays a conserved role in the development of organisms from flies to humans. Spontaneous calcium oscillations can be found in nearly every cell type and tissue, and disruption of these oscillations leads to defects in development. However, the mechanism by which bioelectricity regulates development is still unclear. Ion channels play essential roles in the processes of cell death, proliferation, migration, and in each of the major canonical developmental signaling pathways. Previous reviews focus on evidence for one potential mechanism by which bioelectricity affects morphogenesis, but there is evidence that supports multiple different mechanisms which are not mutually exclusive. Evidence supports bioelectricity contributing to development through multiple different mechanisms. Here, we review evidence for the importance of bioelectricity in morphogenesis and provide a comprehensive review of the evidence for several potential mechanisms by which ion channels may act in developmental processes.

## Introduction

The process by which a single fertilized egg develops into a multicellular organism is a remarkable feat of biology. For most plants and animals, the fertilized egg must undergo dozens of repeated divisions with various lineages of cells proliferating, migrating, and differentiating at exactly the correct times and places within 3D space to form the specialized tissues and organs of the adult organism. This process requires a vast amount of information to be transmitted and processed for the organism to form correctly, and yet this process occurs in every multicellular species.

Development is robust, with organisms and tissues able to withstand damage or induced errors during development and still ultimately develop correctly. This remarkable ability to develop correctly after perturbation can be seen in the development of twins. In the early stages of mammalian development embryos can be completely split into two, and each half can go on to produce a fully developed organism. This splitting can even occur spontaneously as late as 14 days post-fertilization in human embryos and result in the correct development of a set of twins (Hall, 2003). Thus, the process of development is not simply an unfolding of a single developmental pathway encoded rigidly within genetics. Developing organisms can also correct early damage. The imaginal discs in developing *Drosophila* can regenerate after damage or ablation during early development and go on to form functional adult appendages (Smith-Bolton et al., 2009). Severe morphological abnormalities can be induced in *Xenopus* during early craniofacial development and yet go on to later self-correct (Vandenberg et al., 2012; Pinet et al., 2019). This amazing ability of cells and tissues to respond to environmental changes and develop needed structures can additionally be seen in regenerating organisms. Planarians, zebrafish, *Xenopus*, and axolotls can regenerate entire damaged or amputated organs and limbs (Reddien and Alvarado, 2004; Roy and Gatien, 2008; Gemberling et al., 2013; Phipps et al., 2020). An extreme example can be seen in *Hydra vulgaris* (freshwater polyps) which are able to completely reaggregate and regenerate from dispersed cells in suspension (Gierer et al., 1972). This extraordinary ability of tissues and cells to develop correctly even when facing environmental perturbations raises one of the fundamental questions of developmental biology: how do cells communicate and coordinate in a tissue-wide manner to guide development? How does each cell know when and where to proliferate, migrate, and differentiate even in the face of perturbation?

Much of this tissue wide coordination is attributed to the morphogen signaling pathways. These morphogens, including members of the bone morphogenetic protein (BMP) pathway, Wnt pathway, Hedgehog pathway, are secreted proteins that form a concentration gradient across tissues, giving cells positional information based on the concentrations of the various morphogens. According to the morphogen concentration hypothesis, the precise concentration of each of these morphogens activates various genetic pathways that tell each cell what type of cell to differentiate into and where to differentiate (Rogers and Schier, 2011). While morphogen signaling and other canonical signaling pathways (such as Notch signaling) help explain how cells can communicate with each other across space, there is still much that is not understood about how cells precisely coordinate the spatial distribution as well as timing of cellular processes required for development. How exactly morphogen gradients are regulated is a growing question in the field, as the passive diffusion model does not adequately explain gradient formation. Recently, there has been growing evidence that in addition to the classic molecular developmental signaling pathways that cells use to coordinate development, cells use electrical signaling via ion channels to communicate (Harris, 2021; Levin, 2021). This field of research, known as developmental bioelectricity, is growing rapidly. Here, we review the evidence that ion channels are important for development in humans and other organisms, the potential mechanisms by which ion channels may be regulating development, and the next steps and barriers within the field of developmental bioelectricity.

## Overview of Bioelectricity in Development

Ion channels sit within the cell membrane or organelle membranes of the cell and help regulate the levels of calcium, sodium, potassium, chloride, and other charged molecules within the cytoplasm and the other compartments in the cell. The difference in ion concentrations within the cell and in the extracellular space creates a transmembrane potential (V_mem_). All cells have a resting membrane potential, but the exact value of V_mem_ varies by cell type. In general, differentiated cells are hyperpolarized relative to stem cells (Sundelacruz et al., 2009). Rapid changes in V_mem_ are essential for the functioning of excitable cells such as neurons and myocytes as these changes induce the release of neurotransmitters or contraction of muscle cells. In excitable cells these rapid changes are called action potentials. Action potentials occur when an influx of positively charged sodium depolarizes the cell, activating voltage-gated calcium channels that mediate calcium entrance. The influx of calcium activates calcium-sensitive proteins that then mediate neurotransmitter containing vesicle fusion. Eventually, potassium channels open to allow an efflux of positively charged potassium to repolarize the membrane.

**FIGURE 1:**
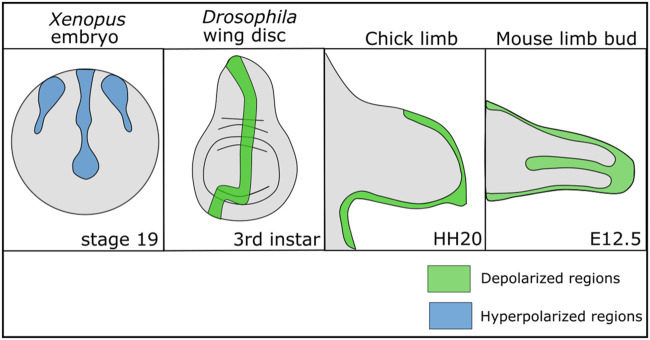
Patterns of depolarization and hyperpolarization across various developing tissues. Developing *Xenopus* embryos have patterns of hyperpolarization across the ectoderm during neurulation (Vandenberg et al., 2011; Adams et al., 2016). In *Drosophila melanogaster*, the developing wing disc at the third instar stage has a stripe of depolarized cells along the anterior posterior boundary (Emmons-Bell and Hariharan, 2021). In developing chick and mouse limbs the mesenchyme is depolarized during chondrogenic differentiation (Atsuta et al., 2019).

While non-excitable cells do not exhibit the same rapid changes in V_mem_ that exitable cells do, there is growing evidence that there are V_mem_ patterns and slow changes in V_mem_ across developing cells that influence development (Levin, 2014; Harris, 2021; Levin, 2021). Regions of relatively depolarized and hyperpolarized cells have been found within multiple different developing organisms from flies to mammals, suggesting that V_mem_ patterns play a conserved role in development (Figure 1). In the *Drosophila* larval wing disc, a stripe of cells along the anterior posterior (A/P) boundary is depolarized relative to the other cells and this V_mem_ pattern is important for wing development (Emmons-Bell and Hariharan, 2021). In developing mouse and chick limb buds, regions of the limb undergoing chondrogenesis shift from a relatively hyperpolarized state to a relatively depolarized state over time as chondrogenesis occurs (Atsuta et al., 2019). Disruption of this depolarized state hinders chondrogenesis (Atsuta et al., 2019). A similar patterning in V_mem_ can be found in developing *Xenopus* embryos, where dynamic regions of hyperpolarized and depolarized cells in the ectoderm change throughout development (Vandenberg et al., 2011). Clusters of hyperpolarized cells in developing *Xenopus* mark the developing eyes, and perturbation of this pattern disrupts eye formation (Pai et al., 2012). In developing mouse and chick limb buds, regions of the limb undergoing chondrogenesis shift from a relatively hyperpolarized state to a relatively depolarized state over time as chondrogenesis occurs (Atsuta et al., 2019). These studies suggest that V_mem_ may play a role in development and a wide number of studies confirm that disrupting ion channels—which collectively control the V_mem_—leads to developmental defects.

## Ion Channel Signaling in Human Development

Ion channel mediated electrical signaling is essential for the proper development of many organisms ranging from planarians to humans. In humans, a set of syndromes known as channelopathies are associated with mutations in ion channel genes. Most of these channelopathies lead to defects in the functioning of the heart or the brain, consistent with the known important roles of ion channels in those organs. However, many of these channelopathies are also associated with morphological defects, suggesting that ion channels play a role in the development of organs and tissues that is not limited to those tissues traditionally associated with ion channel function such as the brain. For example, Andersen-Tawil Syndrome, a channelopathy associated with a mutation in the inwardly rectifying potassium channel Kir2.1, leads to multiple morphological and craniofacial defects including short stature, low-set ears, small-lower jaw, cleft palate, clinodactyly, and syndactyly (Tawil et al., 1994; Plaster et al., 2001; Perez-Riera et al., 2020). Disruption of the homologous channel in *Xenopus*, *Drosophila melanogaster*, and mice also leads to developmental defects within those organisms, suggesting a conserved role for Kir2.1 in development (Dahal et al., 2012; Adams et al., 2016; Dahal et al., 2017; Belus et al., 2018). Timothy Syndrome, caused by gain-of-function mutations in the calcium channel Ca_v_1.2, is associated with multiple developmental defects including fusion of the digits of the hands or feet (syndactyly) and craniofacial defects in humans (Splawski et al., 2004; Splawski et al., 2005). Mouse and zebrafish models of Timothy Syndrome recapitulate the craniofacial defects, suggesting a conserved role for Ca_v_1.2 in development (Ramachandran et al., 2013). Other human syndromes that are associated with ion channel mutations and that lead to morphological defects include Temple-Baraitser Syndrome, associated with mutations in the voltage-gated potassium channel KCNH1, Birk-Barel Syndrome, associated with mutations in the two-pore domain potassium channel KCNK9, Keppen-Lubinsky Syndrome, associated with mutations in the inwardly rectifying potassium channel KCNJ6, and CLIFAHDD Syndrome, associated with mutations in the sodium leak channel NALCN (Barel et al., 2008; Chong et al., 2015; Masotti et al., 2015; Simons et al., 2015). Each of these syndromes lead to various craniofacial and digital defects (Barel et al., 2008; Chong et al., 2015; Masotti et al., 2015; Simons et al., 2015).

Outside of syndromic channelopathies there is additional evidence that disruption of ion channel function can lead to developmental defects within specific tissues and organs. For example, cystic fibrosis is caused by disruption of the epithelial chloride channel cystic fibrosis transmembrane regulator (CFTR) (Davies et al., 2007). Disruption of CFTR leads to a reduced ability of the lungs to clear bacteria and the production of viscous mucus that disrupts proper lung functioning (Davies et al., 2007). Recent evidence, however, also indicates that CFTR is required for proper development of the lungs (Larson and Cohen, 2005; Amaral et al., 2020). CFTR is expressed at very high levels during fetal lung development, and patients with cystic fibrosis present with abnormal lung development as early as 17–19 weeks gestation (Gosden and Gosden, 1984; Larson and Cohen, 2005). In CFTR knockout mice, transient expression of a normal copy of CFTR *in utero* rescues lethality and some of the lung and intestinal phenotypes of cystic fibrosis even when this CFTR is no longer expressed after birth, suggesting that CFTR is particularly important during development (Larson et al., 2000a). Overexpression of CFTR in both mice and primates leads to increases in proliferation and differentiation of fetal lung secretory cells, suggesting that CFTR contributes to timing of proliferation and differentiation within the lung during development (Larson et al., 2000a; Larson et al., 2000b). The identification of multiple human syndromes and developmental defects associated with ion channel disruption is just one of the many lines of evidence suggesting that ion channels are essential for development.

## Conservation of Ion Channel Roles in Morphogenesis

Disruption of ion channel function has been associated with developmental defects within many non-human organisms, suggesting that ion channels play a conserved role in morphogenesis. Dozens of ion channel mutations impacting ion channels of nearly every category have been linked to developmental defects in worms, flies, frogs, fish, and mice (Pai et al., 2012; George et al., 2019; Srivastava et al., 2021). Ion channels are important for regulating both the size and patterning of various tissues. In zebrafish, mutations in the potassium channel gene *kcnk5b* lead to enlarged fins while mutations in the gap junction gene *connexin43* lead to shorter fins, suggesting that ion channel signaling is important for regulating the growth and proportional size of the fins (Iovine et al., 2005; Perathoner et al., 2014; Daane et al., 2018). Mutations in many different calcium, potassium, sodium, and chloride channels in *Caenorhabditis elegans* have been associated with changes in body length or girth, suggesting that ion channels also regulate body size in *C. elegans* (Srivastava et al., 2021). In *D. melanogaster* a screen of wing development identified 44 ion channels important for regulating both wing size and vein patterning, suggesting bioelectrical signaling is important for the overall development of the wing (Dahal et al., 2012; George et al., 2019). Loss of Kir2.1 in mice, the inwardly rectifying potassium channel associated with Anderson-Tawil Syndrome in humans, causes craniofacial and digital defects, suggesting that Kir2.1 is important for patterning of those structures (Dahal et al., 2012; Belus et al., 2018). Injection of a dominant-negative form of the inwardly rectifying potassium channel Kir2.1 in frogs also leads to abnormal craniofacial development (Adams et al., 2016). These craniofacial defects are recapitulated by expression and activation of a light-activated cation channel or a light activated hydrogen pump, suggesting that the role of ion channel function in craniofacial development is not limited to Kir2.1 (Adams et al., 2016). The myriad of ion channels that contribute to morphogenesis in organisms ranging from worms to humans supports the hypothesis that bioelectricity plays an important role in guiding development.

Interestingly, ion channels are important for the establishment of anterior-posterior polarity and tissue identity. For example, trunk fragments of planarians (planarians with both tail and head amputated) usually regenerate both the head and the tail at the proper ends. However, brief treatment of trunk fragments with 8-OH, a gap junction inhibitor, can lead to the regeneration of two heads, creating double-headed animals (Durant et al., 2017). This change in the body axis plan appears to be permanent, with double-headed flatworms continuing to generate two heads after subsequent amputations even when all 8-OH has been removed (Durant et al., 2017). Treating planarian trunk fragments with ionophores to alter the resting membrane potential and depolarize the cells also results in the regeneration of double-headed organisms, suggesting that it is the depolarization of cells that regulates the development of the body axis (Durant et al., 2019). In *Xenopus* embryos, injection of mRNA encoding a dominant-negative form of the potassium channel Kir6.1 was sufficient to induce the formation of ectopic eyes, suggesting bioelectricity can regulate tissue identity as well (Pai et al., 2012). Together, these data strongly support the importance of bioelectrical signaling in development, including regulation of the body axis, regulation of body and body part size, and regulation of patterning.

## Spontaneous Calcium Oscillations in Non-Excitable Tissues

Ion channels that conduct sodium, potassium, calcium, and chloride can influence the levels of cytoplasmic calcium. For example, voltage-gated calcium channels open in response to a depolarized membrane potential. Calcium release from the endoplasmic reticulum is regulated in part by ion channels that conduct other ions. Interestingly, spontaneous calcium oscillations in developing tissues exist in diverse organisms. Excitable cells such as neurons, muscle cells, or pancreatic beta cells communicate and perform their functions through rapid changes in intracellular concentrations of ions to generate action potentials. While most other cells do not propagate action potentials in the same way, many different cells and tissues propagate calcium transients and waves. Calcium waves that propagate spontaneously or in response to stimuli have been found in mesenchymal stem cells (Kawano et al., 2002), chondrocytes (Kono et al., 2006), osteoblasts (Godin et al., 2007), keratinocytes (Tsutsumi et al., 2009), endothelial cells (Uhrenholt et al., 2007; Yokota et al., 2015; Justet et al., 2019), and epithelial cells (Nathanson, 1994; Evans and Sanderson, 1999; Nihei et al., 2003). These calcium oscillations are due to rapid changes in cytosolic calcium. Calcium is stored in the endoplasmic reticulum and the mitochondria. Calcium channels and gap junctions located in the cell membrane as well as channels in the endoplasmic reticulum have been found to play a key role in the propagation of spontaneous calcium waves (Uhlén and Fritz, 2010). Calcium levels in the cytosol rise when calcium is brought across the cell membrane by gap junctions or activated-voltage gated calcium channels, or when the ER stores of calcium are released into the cytoplasm (Uhlén and Fritz, 2010). This increase in cytosolic calcium is then brought back down either by movement of the calcium through gap junctions into other cells, by being pumped back into the ER through the ATPase SERCA, being pumped out of the cell, or by being taken up by mitochondria (Uhlén and Fritz, 2010). The change in calcium levels between the cytosol, the ER, and mitochondria lead to the propagation of the calcium oscillations that are observed in cells (Uhlén and Fritz, 2010). The function of these dynamic changes in calcium is not well understood. Recently, however, these calcium waves and transients have been found within wide number of developing tissues, and disruption of the calcium oscillations disrupt morphogenesis, suggesting that calcium dynamics may help coordinate development.

Disruption of these dynamic changes in calcium in some tissues can lead to abnormal development. In *D. melanogaster*, the larval developing wing epithelium propagates calcium waves both in response to wounding and spontaneously *in vivo* (Narciso et al., 2015; Restrepo and Basler, 2016; Balaji et al., 2017; Brodskiy et al., 2019). Disruption of these calcium waves either pharmacologically or through mutations impacting ion channels required for these calcium waves is associated with disruption in proper *Drosophila* wing development (Brodskiy et al., 2019). Blue pansy butterflies also spontaneously propagate calcium waves and transients during pupal wing development and disruption of these oscillations leads to malformed scale-development and eye spot formation in the wing (Ohno and Otaki, 2015). In these blue pansy butterflies, many of the calcium oscillations appear to originate from the future eye spot of the developing wing, suggesting that the calcium oscillations may instruct development of this structure in the wing (Ohno and Otaki, 2015). Calcium oscillations in developing tissues are not limited to invertebrates (Slusarski and Pelegri, 2007). Calcium oscillations have been found in budding chick feather buds and inhibition of these calcium oscillations disrupts cell migration and feather bud formation (Li et al., 2018). Calcium oscillations occur in cultured primary mouse embryonic palate cells (Isner and Bates, 2021) and they have also been imaged during early embryonic development in zebrafish (Webb and Miller, 2006), *Xenopus* (Wallingford et al., 2001), and mouse embryos (reviewed in Stewart and Davis, 2019). The existence of these calcium oscillations within many different organisms and tissues during development paired with the evidence that blocking them leads to developmental defects suggests that calcium oscillations are important for development.

The growing understanding of the role of spontaneous calcium oscillations and bioelectrical signaling in non-neuronal cells is reminiscent of what is known about the evolutionary development of synapses and neural connections. Research into the potential origins of neuronal synapses suggests that many synaptic proteins likely originated in non-neuronal cells before being co-opted by neurons (Ovsepian and Vesselkin, 2014; Ovsepian, 2017; Ovsepian et al., 2020). The potential use of calcium oscillations and bioelectrical signaling in the development of non-neuronal tissues may support the hypothesis that this rudimentary bioelectrical signaling may have evolved over time to help develop the fine-tuned chemical synapse in neural connections (Ovsepian and Vesselkin, 2014; Ovsepian, 2017; Ovsepian et al., 2020).

## Ion Channels in Development: Potential Mechanisms of Action

While it is becoming increasingly evident that ion channels are essential for proper development, the mechanism by which they act is still unclear. How do cells within tissues and organs use bioelectricity during development?

Recent studies suggest that it is the differences in the V_mem_ across cells that is important for development, rather than specific ion channels or ions. For example, in *Xenopus laevis* disruption of the homolog of Kir2.1, the channel associated with Anderson-Tawil Syndrome in humans, leads to craniofacial defects (Adams et al., 2016). These defects were recapitulated by optogenetic activation of a non-specific cation channel or optogenetic activation of a hydrogen pump, both expected to cause similar changes in the V_mem_ as Kir2.1 disruption (Adams et al., 2016). In contrast, disruption of a sodium-hydrogen exchanger that was expected to be electroneutral and cause no change in V_mem_ led to no disruption of craniofacial development (Adams et al., 2016). In *Xenopus* embryos the development of ectopic eyes can be induced by the injection of ion channel expression constructs that depolarize regions of the embryo (Pai et al., 2012). This induction of ectopic eyes is not limited to a single ion channel construct, however, and a variety of different constructs lead to the same ectopic eye formation (Pai et al., 2012). These results suggests that it is the overall V_mem_ which is collectively controlled by ion channels—rather than the specific identity of the channels or ions—that is important for morphological development (Adams et al., 2016).

While it is clear that V_mem_ is important for development, less is known about the downstream molecular mechanism by which bioelectricity regulates morphogenesis. There are several potential mechanisms by which bioelectricity may play a role in development. Calcium is required for various cellular processes that feed directly into development. What lessons can we learn from excitable cells, like neurons and pancreatic beta cells? In these cells, sodium, potassium, and chloride channels determine V_mem_. Several calcium channels open or close in response to a particular Vmem. Thus, channels that do not conduct calcium contribute to intracellular calcium concentration. Could V_mem_ play a central role as a regulator of calcium, which mediates proliferation, apoptosis, cell cycle control, cell polarity, cell migration, and even molecular signaling? Evidence for each of these potential mechanisms is described below.

## Cell Death Pathways

**FIGURE 2:**
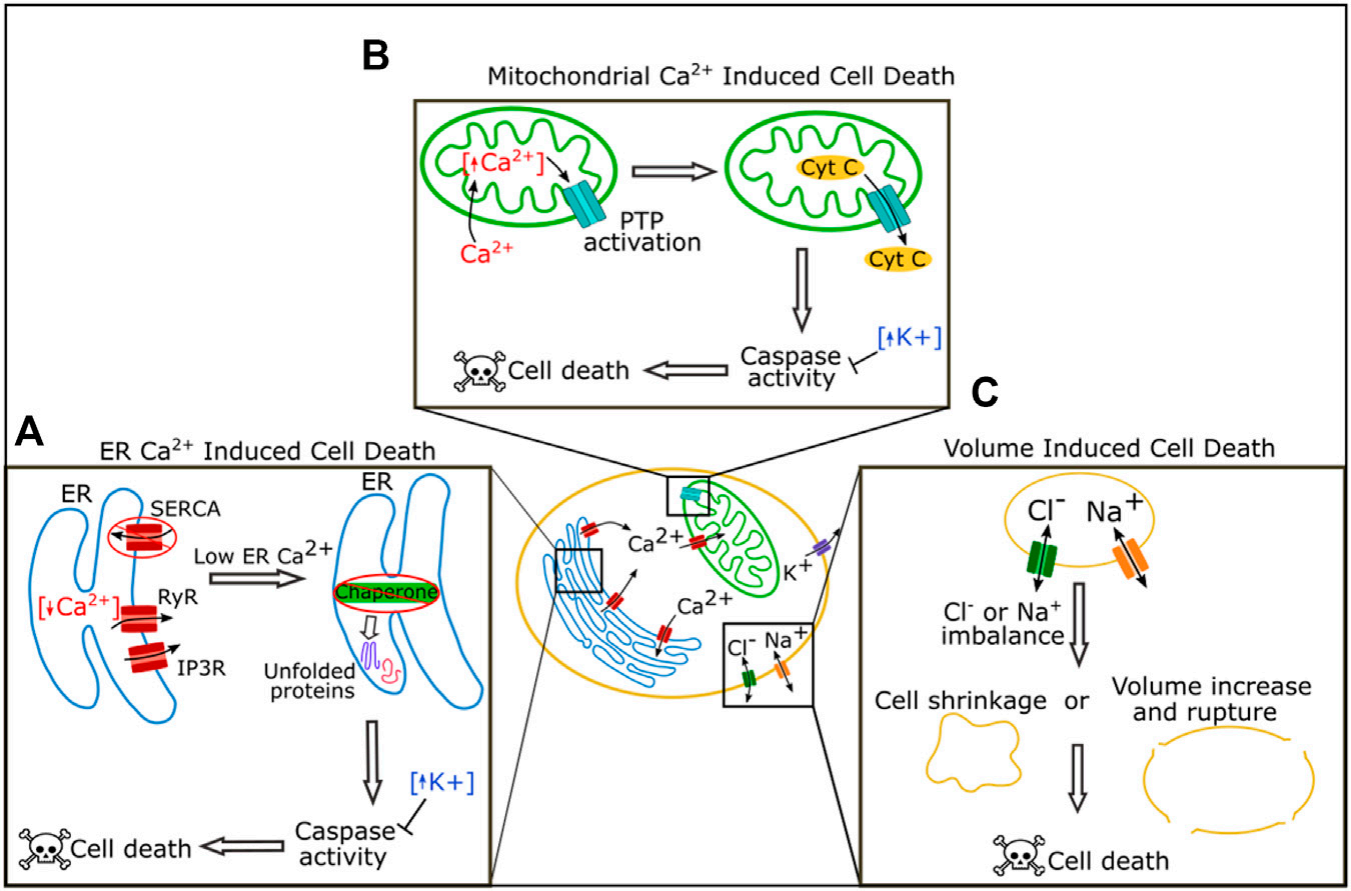
Roles of ion channels in cell death regulation. In the ER Reduction of calcium by blockage of SERCA or increased activity of the receptors RyR and IP3R can lead to loss of chaperone function triggering the unfolded protein response pathway, caspase activation, and ultimately cell death **(A)**. In the mitochondria, increased levels of calcium lead to activation of the permeability transition pore (PTP) which can cause leakage of cytochrome C and ultimately cause cell death **(B)**. Chloride and sodium both regulate cell death by regulating cell volume. Extreme imbalance of chloride or sodium levels leads to cell shrinkage or volume increase and rupture, and both cause cell death **(C)**.

Ion channels play an important role in cell death pathways including both apoptosis and necrosis (Figure 2, and reviewed in Lang et al., 2006; Bortner and Cidlowski, 2014). Potassium leaves the cell during early apoptosis leading to a depletion of intracellular potassium (Bortner et al., 1997; Yu, 2003). This loss of potassium is important for the apoptotic pathway and inhibition of potassium efflux can prevent apoptosis (Bortner et al., 1997; Yu, 2003). Potassium is one of the most abundant ions within the cell and physiological concentrations of potassium have an inhibitory effect on caspase and nuclease activity (Hughes et al., 1997; Dallaporta et al., 1998). A number of potassium channels have been identified as playing an important role in mediating apoptosis. These include outward delayed rectifier (IK) channels, voltage-gated potassium channels, the inward rectifier Kir1.1, and multiple calcium activated potassium channels (reviewed in Yu, 2003). Drops in potassium levels lead to shrinkage of the cell due to changes in osmolarity, and this shrinkage may contribute to apoptosis (Yu, 2003). While lowering potassium concentration by itself is not sufficient to induce apoptosis, potassium depletion facilitates apoptosis and may be a universal part of the apoptotic pathway (Hughes et al., 1997; Dallaporta et al., 1998; Yu, 2003).

Calcium acts as a key signaling molecule in apoptosis. Calcium is stored at high concentrations in the ER and the mitochondria, and at a lower concentration in the cytosol. An imbalance of these calcium stores can lead to cell death (Figure 2A, reviewed in Orrenius et al., 2003; Rizzuto et al., 2003; Zhivotovsky and Orrenius, 2011). Calcium was first associated with cell death when it was found that cells killed by withholding oxygen or treating with a cytotoxic drug had a dramatic rise in calcium content (Chien et al., 1977; Schanne et al., 1980). Cytosolic calcium increases during apoptosis (Martikainen et al., 1991; Kruman et al., 1998), and overactivation or disruption of calcium channels increases cell death. Disruption or overactivation of the ER calcium regulating channels inositol 1,4,5-trisphosphate (IP3) receptors (IP3Rs), ryanodine receptors (RyRs), and SERCA all increase cell death (Ruiz et al., 2010; Kiviluoto et al., 2012; Sehgal et al., 2017).

There are multiple pathways by which an imbalance in calcium levels can induce cell death (Figures 2A,B). One pathway is via the induction of prolonged ER stress (Figure 2A). The ER is an essential organelle required for protein folding and processing (Adams et al., 2019). Inside the ER, a wide variety of chaperones help process and fold new proteins, and many of these chaperones require calcium to function correctly (Adams et al., 2019). If levels of calcium in the ER drop, the ability of chaperones to efficiently fold proteins is reduced, and misfolded or unfolded proteins accumulate, a situation known as ER stress (Adams et al., 2019). ER stress initially induces the unfolded protein response (UPR) pathway (Adams et al., 2019). Long term chronic ER stress, however, leads to the expression or activation of C/EBP-homologous protein (CHOP), c-Jun N-terminal kinase (JNK), caspases, and other pro-apoptotic proteins, to induce apoptosis (Nakagawa et al., 2000; Hiramatsu et al., 2015).

High intracellular calcium can induce cell death through mitochondria (Figure 2B). Mitochondria take up calcium from the cytoplasm. Sharp rises in cytoplasmic calcium can overload calcium in the mitochondria which induces the opening of the permeability transition pore (PTP), a complex in the inner mitochondrial membrane (Williams et al., 2013; Bonora and Pinton, 2014). Extreme PTP activation causes swelling and rupture of the mitochondria resulting in necrosis, while milder activation of PTP can lead to leakage of cytochrome C and the induction of apoptosis (Bonora and Pinton, 2014).

Changes in sodium and chloride flux have also been reported during apoptosis (Figure 2C). Sodium levels increase within the cell during apoptosis, and activation of voltage-gated sodium channels (VGNCs) via the VGNC activator veratridine can induce apoptosis in neurons (Dargent et al., 1996; Koike et al., 2000; Arrebola et al., 2005). Chloride flux is important for apoptosis and blocking chloride channels can block apoptosis, perhaps because this ion regulates cell volume (Okada et al., 2004; Okada et al., 2006).

Ion channels play an important role in cell death pathways, and mediation of apoptosis is one mechanism by which bioelectricity influences development.

## Proliferation and Cell Cycle Regulation

Ion channels play help regulate cell proliferation. The transmembrane potential of cells changes over the course of the cell cycle (Sachs et al., 1974; Wonderlin et al., 1995). It was observed as early as the 1970s that depolarization could induce mitosis of neuronal precursors (Stillwell et al., 1973; Cone and Cone, 1976). It is now known that calcium, potassium, sodium, and chloride all play roles in regulating the cell cycle (Blackiston et al., 2009).

**FIGURE 3:**
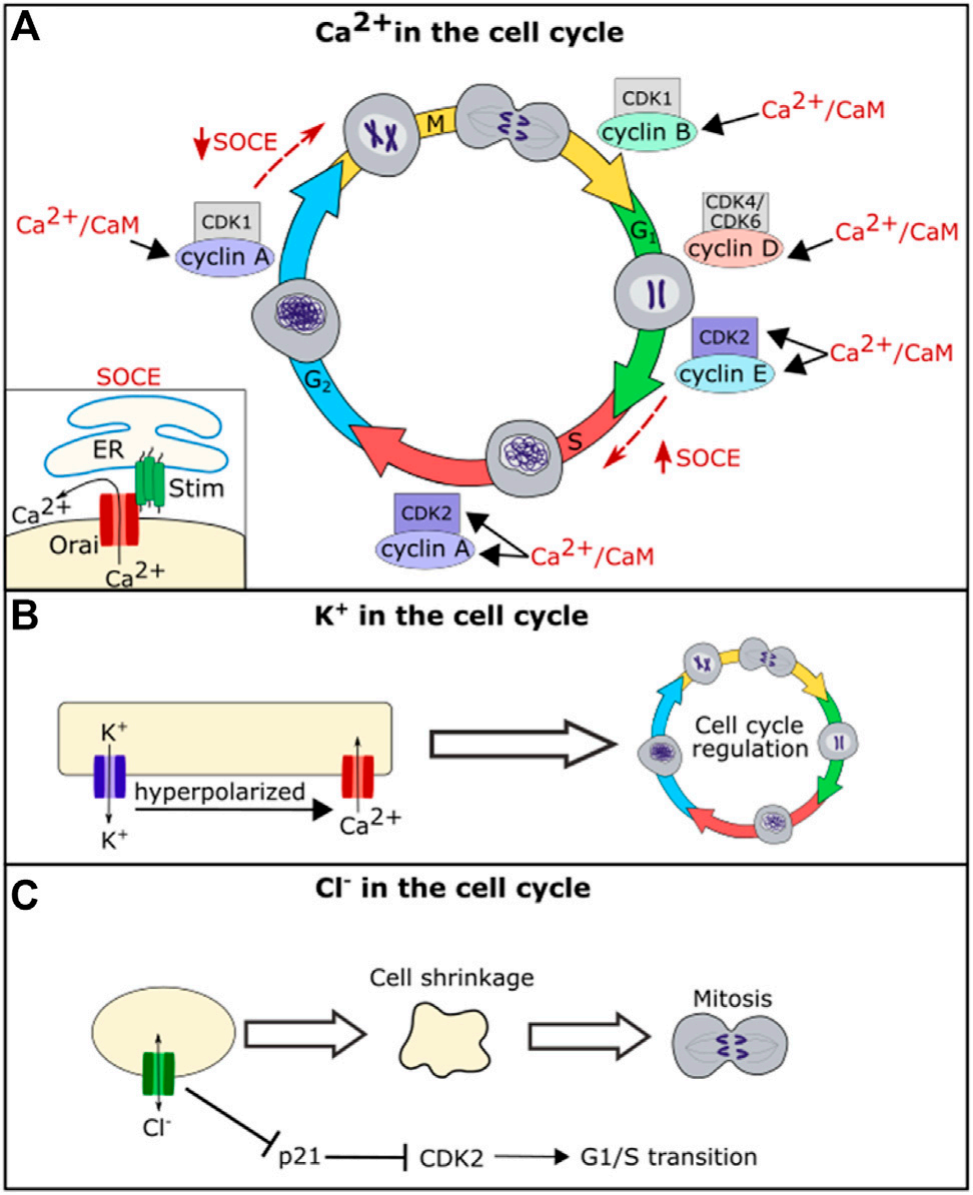
Schematic diagram of the role of ion channel function in cell cycle regulation. Ca^2+^/CaM regulates levels of CDK2, cyclin A, cyclin B, cyclin D, and cyclin E **(A)**. Calcium further regulates the cell cycle through the store-operated calcium entry (SOCE) Pathway. SOCE is upregulated at the G1/S phase transition and downregulated at the G2/M phase transition **(A)**. Potassium flux hyperpolarizes the cell, which helps drive calcium into the cell regulating calcium influence on the cell cycle **(B)**. Chloride flux is required for the cell shrinkage that is necessary for mitosis and also regulates levels of p21 **(C)**.

Calcium plays an essential role in regulating the cell cycle at nearly every transition step (Humeau et al., 2018) (Figure 3). The cell cycle is controlled by cyclin-dependent protein kinases (CDKs) that activate upon binding to a cyclin. Expression of each of the cyclins regulates the CDK complexes and guides entry into the next phase of the cell cycle. Calcium feeds into the cell cycle primarily by regulating calmodulin (CaM) and calcineurin (CaN) (Humeau et al., 2018). CaM is a protein that is activated upon binding of calcium. Ca^2+^/CaM can directly regulate CDKs and cyclins or act through activation of calcineurin, a phosphatase that activates upon Ca^2+^/CaM binding (Kahl and Means, 2003). Together Ca^2+^/CaM and Ca^2+^/CaM activated-calcineurin regulate many of the CDKs and cyclins (Kahl and Means, 2003). For example, calcium acts through CaM or CaN activation to regulate the levels CDK1, CDK2, cyclin A, cyclin D, and cyclin E within various cell types (Colomer et al., 1994; Tomono et al., 1998; Kahl and Means, 2004; Humeau et al., 2018) (Figure 3).

In addition to acting through CaM and its downstream pathways, calcium oscillations are important for regulating cell cycle phase transitions. Calcium oscillations have been found to be important for the G_1_/S phase transition. The store-operated calcium entry (SOCE) pathway, a pathway in which the calcium channels Stim and Orai work to bring calcium into the cell upon ER calcium depletion, was found to be upregulated during G_1_/S phase transition and downregulated prior to the G2/M phase transition (Chen et al., 2016) (Figure 3). Blocking SOCE can lead to G1 arrest (Short et al., 1993; Chen et al., 2016). Treating cells with calcium blockers can also lead to inhibition of the metaphase-anaphase transition, suggesting that calcium helps regulate the mitotic spindle checkpoint in addition to the G_1_/S phase transition (Xu et al., 2003). Together these data suggest that calcium is an important second messenger for regulating both cell death and cell life pathways.

There is a large body of evidence that indicates that potassium also plays an important role in cell-cycle regulation (Urrego et al., 2014). Treating cells with potassium channel blockers can block proliferation (DeCoursey et al., 1984; Amigorena et al., 1990; Lee et al., 1993), and this is partially due to potassium’s role in calcium movement. The potassium gradient hyperpolarizes the cell membrane, driving calcium entry into the cell and thus potassium can regulate the cell cycle via the calcium mediated pathways described above (Urrego et al., 2014). However, the role of potassium channels in hyperpolarizing the cell membrane is not the only mechanism by which they influence the cell cycle. Multiple potassium channels including K_V_1.3, K_V_3.1, and K_V_10.1 impact the cell cycle even when they are modified to prevent ion permeation, suggesting that they may interact with cell signaling via a mechanism that is independent of potassium conduction (Downie et al., 2008; Millership et al., 2011; Cidad et al., 2012; Urrego et al., 2014).

While chloride appears to play a less important role in the cell cycle than calcium and potassium, the chloride channel ClC3 plays a role in cell cycle regulation in nasopharyngeal carcinoma cells and in glial cells, with disruption of CIC3 inhibiting cell proliferation (Habela et al., 2008; Xu et al., 2010). ClC3 regulates cell volume, and chloride efflux is necessary to cause the reduction in volume seen in mitotic cells (Habela et al., 2009). A reduction in cell volume via efflux of salt and water is important for mitosis, and cells that are forced to maintain a larger volume take longer to divide (Habela and Sontheimer, 2007). When chloride flux is blocked, cells cannot reduce their volume prior to mitosis, and this leads to a delay in cell division. Intracellular chloride levels have also been shown to directly regulate the cell cycle by regulating the expression level of p21. Loss of chloride leads to an upregulation of p21 which in turn leads to a downregulation of CDK2 and cell cycle arrest at the G_1_/S cell cycle checkpoint (Miyazaki et al., 2008; Shiozaki et al., 2011). Changes in proliferation because of disruption of ion channel function in individual cells would impact the size of a whole tissue.

The role of ion channels in proliferation as well as cell death pathways is one of the primary reasons that ion channel mutations are common in cancer cells. The role of ion channels in cancer has been reviewed extensively (Lang and Stournaras, 2014; Prevarskaya et al., 2018). The variety of ion channels linked to cancer suggest that ion channels play an important role in regulating the cell cycle.

## Cell Polarity and Migration

Ions are important for both the establishment of cell polarity and the progression of cell migration (Campetelli et al., 2012). In human bone osteosarcoma U2OS cells, it has been observed that many calcium channels, including those that regulate the ER stores of calcium, tend to concentrate at the rear end of polarized cells (Huang et al., 2015). Disruption of a variety of calcium channels using drugs that targeted transient receptor potential channels (TRPC), calcium release activated channels (CRAC), or store-operated calcium entry (SOCE) channels, all led to a decrease in cell polarization (Huang et al., 2015). Disruption of STIM via knockdown or expression of a dominant-negative form of STIM, similarly reduced cell polarization (Huang et al., 2015). While the mechanism by which calcium channels regulate cell polarity is unclear, at the immune synapse calcium organizes actin filament formation (Hartzell et al., 2016). Actin plays an essential role in planar cell polarity, and it is possible that calcium impacts cell polarity by regulating actin dynamics.

The establishment of cell polarity is essential for the migration of cells. Calcium plays roles in cell migration *in vivo* as well as in cell culture. For example, blocking spontaneous calcium waves in the developing chick feather bud disrupts normal cell migration leading to malformed feather buds (Li et al., 2018). Calcium is similarly important for the migration of zebrafish primordial germ cells (PGCs) (Blaser et al., 2006). In these PGCs it was found that calcium levels increased at the front of migrating cells and this increase was necessary for proper migration (Blaser et al., 2006). Blaser et al. hypothesize that this increase in calcium may activate acto-myosin contraction, directing cell migration (Blaser et al., 2006). Cell migration is important for morphogenesis of several structures. If individual cells cannot migrate properly due to inhibition or loss of ion channel function, cells will not be in the right place at the right time to send or receive developmental signals and the tissue would not develop normally. In addition, lack of effective migration could prevent the correct number of cells from reaching their proper location in a tissue. Therefore, disruption of ion channels could impact the development of a structure by hindering cellular migration.

Other ions contribute to establishment of cell polarity. Inhibition of Na,K-ATPase or treatment with a sodium ionophore in epithelial cells leads to a loss of cell polarity, suggesting that regulation of sodium is important for establishment of cell polarity (Rajasekaran et al., 2001). Overexpression of RhoA GTPase rescues this loss of cell polarity (Rajasekaran et al., 2001). Rho organizes actin and tight junctions in polarized epithelia (Nusrat et al., 1995), so this suggests that sodium is important for this Rho-dependent cell polarization pathway.

## Regulation of Canonical Developmental Signaling Pathways

While ion channels and ions regulate many essential cellular processes long known to be important for development, there is a more recent hypothesis that ion channels may directly regulate the morphogen signaling pathways to coordinate development. Within multiple organisms, loss of ion channel function is associated with disruptions in the BMP signaling pathway, the Notch signaling pathway, the Wnt signaling pathway, and the Hedgehog signaling pathway.

### Bone Morphogenetic Protein Pathway

BMPs are signaling proteins that are essential for the development of organs and tissues, regulating proliferation, apoptosis, and differentiation. Disruption of various ion channels leads to defects in BMP signaling, suggesting that bioelectrical signaling may help regulate this pathway. In mouse bone marrow mesenchymal stem cells (BMSCs), a disruption of BMP signaling and differentiation was found upon knockout of the calcium channel Orai1 (Lee et al., 2016). This disruption of BMP signaling could be rescued by expression of a constitutively active BMP receptor (Lee et al., 2016). Orai1 is a CRAC that helps regulate ER calcium, so loss of BMP signaling upon Orai1 knockout suggests that ER calcium may help regulate BMP signaling in mouse BMSCs. Another channel involved in ER calcium regulation, sarcoendoplasmic reticulum calcium transport ATPase (SERCA) plays a role in the regulation of BMP signaling. In the *D. melanogaster* air sac primordium (ASP), downregulation of SERCA leads to a decrease in BMP/Dpp signal transduction (Huang et al., 2019). Similar impacts on BMP/Dpp signal transduction were found upon knockdown of the voltage-gated calcium channel genes *straightjacket* (*stj*) and *cacophony* (*cac*) (Huang et al., 2019). Interestingly this disruption of BMP/Dpp signal transduction in the ASP was also found upon knockdown of the calcium binding proteins Syt4 or synaptobrevin (Syb) (Huang et al., 2019). Syt4 and Syb are both involved in vesicle trafficking, suggesting that proper BMP/Dpp signaling in this system may require vesicle trafficking mediated by calcium (Huang et al., 2019).

Potassium channels also play a role in BMP/Dpp signaling. Kir2.1 is an inwardly rectifying potassium channel that when disrupted in humans is associated with morphological differences as part of Andersen-Tawil Syndrome. Kir2.1 function is associated with proper BMP signaling in multiple organisms. In mice, Kir2.1 knockout leads to abnormal limb development, craniofacial defects, and a significant reduction in Smad 1/5/8 phosphorylation indicating that Kir2.1 is required for BMP pathway functioning in mammals (Belus et al., 2018). Similar craniofacial defects occur in developing frogs upon loss of Kir2.1 function (Adams et al., 2016). In the *Drosophila* wing disc, loss of function of Irk2, the *Drosophila* ortholog of Kir2.1, reduces downstream phosphorylation of Mad and BMP/Dpp target gene expression (Dahal et al., 2012). The similar developmental disruptions that occur in flies, frogs, and mice upon Kir2.1/Irk2 disruption, suggest that this potassium channel plays a conserved role in development.

**FIGURE 4:**
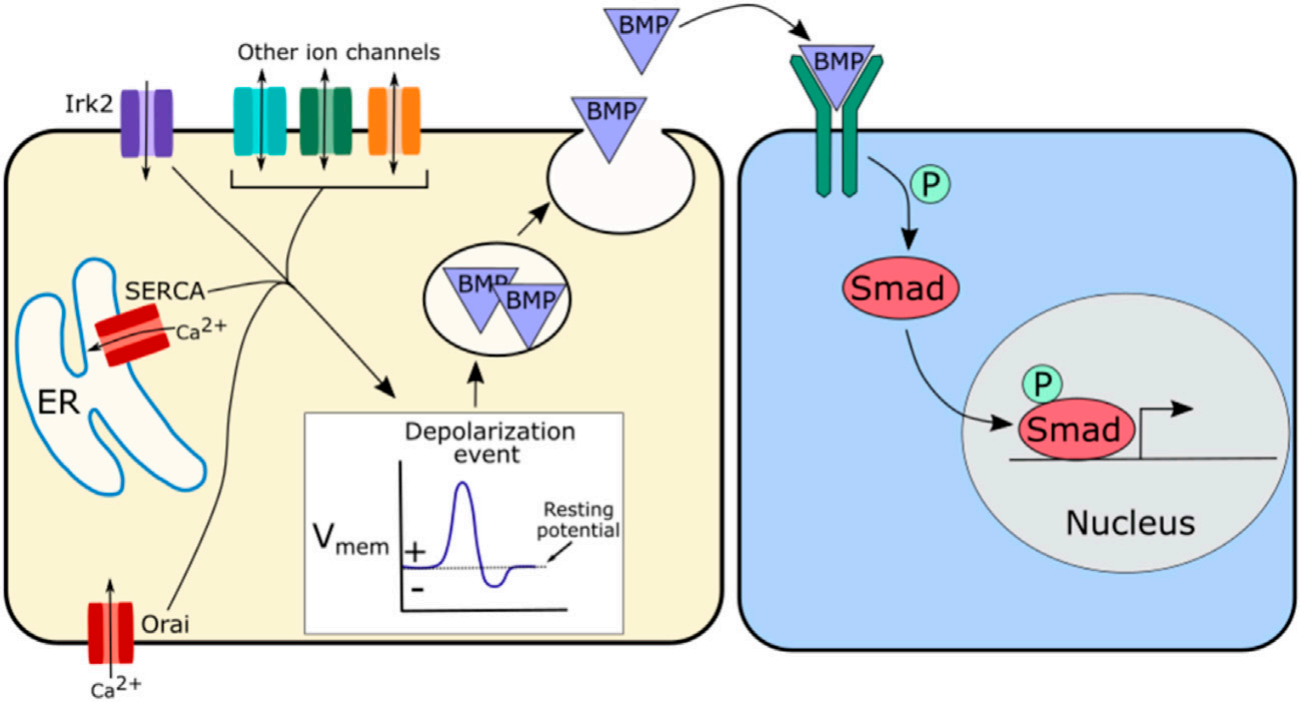
Schematic of potential mechanism by which ion channels may regulate BMP signaling. Irk2, SERCA, and Orai have all been implicated in BMP signaling, but other channels are likely involved as well. A suggested hypothesis is that these ion channels regulate depolarization events that in turn regulate the release of BMP containing vesicles. This regulated release of BMP further regulates BMP pathway activity downstream by modulating the availability of morphogen levels.

In *Drosophila* loss of Irk2 function disrupts BMP/Dpp secretion dynamics, likely leading to the disruption of BMP/Dpp signaling and defects in wing morphogenesis (Dahal et al., 2012; Dahal et al., 2017). Irk2 disruption also abolishes spontaneous calcium oscillations in the wing, so this impact of Irk2 on BMP/Dpp secretion may be mediated through its impact on calcium (Dahal et al., 2012; Dahal et al., 2017). One potential hypothesis is that Irk2 along with calcium channels or other ion channels regulate depolarization events, which in turn regulate the fusion of BMP/Dpp containing vesicles to the cell membrane (Figure 4). This would explain a potential mechanism by which BMP/Dpp secretion could be regulated, impacting propagation of the downstream BMP signaling pathway. Depolarization of the developing *Drosophila* wing evokes BMP/Dpp release, supporting this model (Dahal et al., 2017).

### Notch Pathway

Notch signaling is another canonical developmental signaling pathway that is impacted by the disruption of ion channels. Notch signaling is a conserved signaling pathway required for the development of many tissues and organs (Kopan and Ilagan, 2009). The ligands in the Notch pathway are transmembrane proteins rather than secreted ones and thus Notch signaling acts as a short range signal (Kopan and Ilagan, 2009). Notch signaling regulates cell division, cell death, and cell differentiation (Kopan and Ilagan, 2009).

**FIGURE 5:**
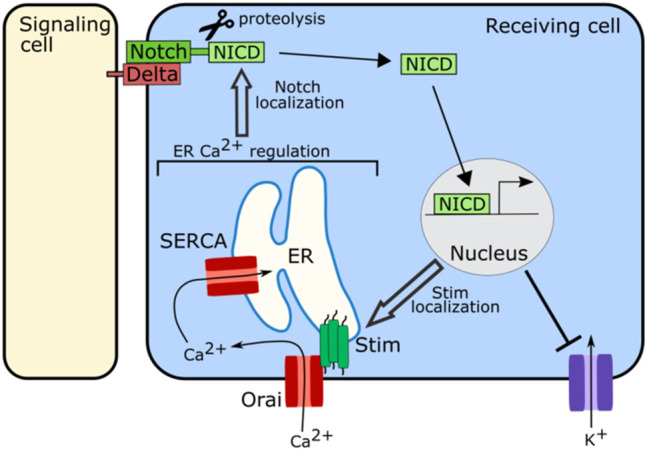
Schematic of role of ion channels in Notch signaling. The ion channels involved in regulating calcium levels in the ER including SERCA, Stim, and Orai, are required for proper localization of Notch at the cell membrane to participate in signaling. Notch signaling in turn regulates the localization and clustering of Stim. Notch signaling also attenuates the activity of potassium channels.

Regulation of ER stores of calcium are important for proper functioning of the Notch signaling pathway (Figure 5). In *Drosophila*, SERCA, a channel that pumps calcium into the ER, is particularly important for Notch signaling. Disrupting SERCA function in *Drosophila* S2 cells, in the *Drosophila* eye, or in the *Drosophila* larval wing disc, leads to developmental defects consistent with a loss of Notch signaling as well as an accumulation of notch and delta receptors in intracellular vesicular structures away from the cell surface (Periz and Fortini, 1999; Suisse and Treisman, 2019). This accumulation of notch away from the cell surface is also seen in the *Drosophila* wing disc when Orai is knocked down (Suisse and Treisman, 2019). Orai and SERCA both act to regulate ER calcium levels, suggesting that ER calcium is important for the trafficking of notch or delta (Figure 5). Loss of SERCA functioning in human leukemia cells, also leads to intracellular accumulation of Notch with the Notch1 receptor failing to fully mature, suggesting that this role of ER calcium in Notch signaling may be conserved (Roti et al., 2013). The Notch receptor contains calcium binding EGF-like repeats, and it is possible that when calcium levels in the ER drop, the notch receptor is no longer able to fold correctly leading to its accumulation within the ER or a failure to traffic correctly to the cell surface (Rand et al., 1997).

Notch signaling also regulates bioelectricity (Figure 5). In human embryonic kidney 293 (HEK293) cells and in myocytes upregulation of Notch signaling has been associated with an increase in cytosolic calcium and decreases in potassium flux (Khandekar et al., 2016; Song et al., 2020). In the HEK293 cells Notch signaling attenuates the activity of voltage-gated potassium channels while also inducing clustering of Stim channels, leading to an influx of calcium into the cytoplasm from the ER (Song et al., 2020). These results suggest that Notch signaling may both regulate and be regulated by ion channel function.

### Wnt Pathway

**FIGURE 6:**
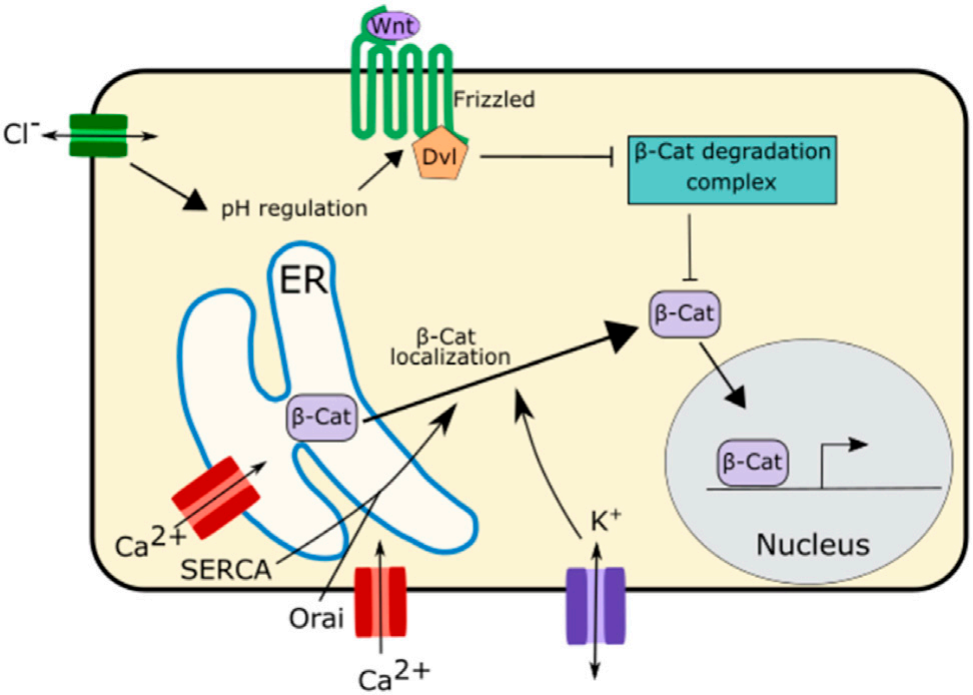
Schematic of mechanisms by which ion channels regulate Wnt signaling. The ER calcium regulating channels SERCA and Orai as well as potassium channels regulate the localization and trafficking of β-Catenin from the ER to the cytoplasm. This enables β-Catenin to participate in Wnt signaling.

Wnt Signaling, another important developmental signaling pathway is also regulated by ion channel function. In the *Drosophila* wing disc disruption of SERCA, an ER calcium channel, causes E-Cadherin to be retained in the ER (Suisse and Treisman, 2019). This causes β-catenin/Arm, which binds to E-Cadherin, to be sequestered in the ER and unable to participate in signaling, leading to downregulation of Wnt signaling (Suisse and Treisman, 2019) (Figure 6). This downregulation of Wnt signaling was also found upon disruption of the ER calcium regulating channel Orai, suggesting that ER calcium plays an important role in Wnt signaling (Suisse and Treisman, 2019) (Figure 6).

While there is evidence that calcium is important for Wnt signaling propagation, potassium and chloride both appear to play even more important roles in this pathway (Rapetti-Mauss et al., 2020) (Figure 6). Potassium regulates the localization of β-catenin impacting Wnt signaling. Inhibition of the potassium channel KCNQ1 downregulates Wnt/β-catenin signaling. This is due to the role of KCNQ1 in regulating the membrane potential. Overactivation of KCNQ1 hyperpolarizes cell membrane while inhibition of KCNQ1 depolarizes the cell membrane (Rapetti-Mauss et al., 2017; Rapetti-Mauss et al., 2020). Inhibition of KCNQ1 and the subsequent depolarization of the membrane inhibits β-catenin from localizing to the cell membrane attenuating Wnt/β-catenin signaling (Rapetti-Mauss et al., 2017; Rapetti-Mauss et al., 2020).

Chloride signaling, too, has been associated with regulation of Wnt/β-catenin signaling. Disruption of CTFR, the chloride channel associated with cystic fibrosis, leads to an increase in intracellular pH (Strubberg et al., 2018). This change in pH enhances the interaction between the Wnt signaling receptors Disheveled and Frizzled leading to an increase in Wnt signaling (Strubberg et al., 2018; Rapetti-Mauss et al., 2020). This increase in Wnt signaling upon CTFR disruption may be one of reasons why cystic fibrosis is associated with abnormal lung development and increased risk of gastrointestinal cancer (Neglia et al., 1995; Larson and Cohen, 2005).

### Hedgehog Pathway

The hedgehog signaling pathway family members, including sonic hedgehog (Shh), desert hedgehog (Dhh), and Indian hedgehog (Ihh) all play an essential role in embryonic patterning and development (Choy and Cheng, 2012). In the hedgehog pathway, the ligands act as secreted morphogens facilitating longer range signaling (Choy and Cheng, 2012). While evidence suggests that calcium can regulate BMP and Notch signaling, in contrast hedgehog signaling appears to primarily act upstream of calcium, regulating calcium oscillations. Recent studies suggest that calcium may play an important role in the execution of the hedgehog signaling pathway. In zebrafish, disruption of intracellular calcium release from the ER via RyRs resulted in abnormal neural tube patterning which was attributed to a loss of Shh-dependent gene expression (Klatt Shaw et al., 2018). RyR function was found to be specifically important for the Shh ligand receiving cells, indicating a role for calcium in Shh signal transduction (Klatt Shaw et al., 2018). Multiple other studies have also implicated spikes of calcium in the execution of Shh induced signaling in *Xenopus*, mouse, and rat embryos and cell lines (Osawa et al., 2006; Heo et al., 2007; Belgacem and Borodinsky, 2011). While the exact mechanism by which Shh induced calcium oscillations modulate gene expression is unclear, it has been suggested that Shh-mediated induction of calcium activates ERK signaling which in turn changes gene expression (Osawa et al., 2006). Shh was also found to mediate calcium oscillations in chick feather buds (Li et al., 2018). In the chick feather bud it was found that Shh could induce expression of the calcium channels *Connexin-43* and *Stim1* to induce calcium oscillations which were important for the migration of the cells in the bud (Li et al., 2018).

**FIGURE 7:**
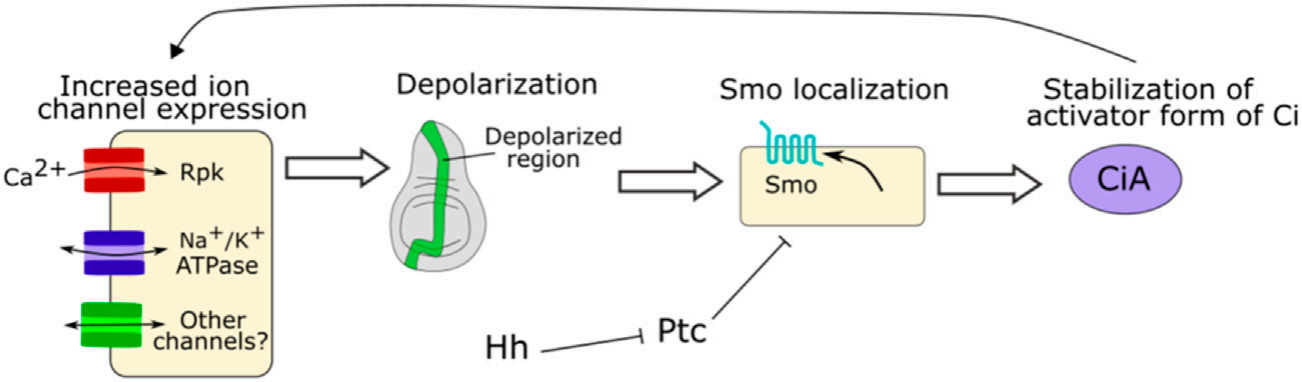
Schematic of role of ion channel function in Hh signaling. In the *Drosophila* wing increased expression of Rpk and Na^+^/K^+^ ATPase and other ion channels generates a depolarized region in the developing wing disc. This depolarization is necessary for proper Smo localization and downstream stabilization of Ci. In turn, Hh signaling regulates levels of Rpk and Na^+^/K^+^ ATPase (Emmons-Bell and Hariharan, 2021).

In the developing *Drosophila* wing (wing disc), hedgehog signaling and ion channel control of V_mem_ mutually reinforce each other (Emmons-Bell and Hariharan, 2021) (Figure 7). A Vmem reporting dye shows a stripe of depolarized cells can be found near the anterior/posterior (A/P) boundary, with the depolarization becoming more restricted to the anterior side of the boundary over time (Emmons-Bell and Hariharan, 2021). Disrupting degenerin epithelial Na^+^ channels (DEG/ENaC) prevents depolarization of that stripe of cells (Emmons-Bell and Hariharan, 2021). Upon both an increase or decrease in hedgehog signaling via activation of a temperature sensitive hedgehog allele or a constitutively active Cubitus interruptus (Ci) allele, the expression levels of the DEG/ENaC channel Rpk and the Na^+^/K^+^ ATPase subunit ATPα were found to change (Emmons-Bell and Hariharan, 2021). These expression levels of Rpk and ATPα correspond with a change in V_mem_, suggesting that hedgehog signaling regulates the Vmem pattern of cells within a tissue via regulation of ion channel expression (Emmons-Bell and Hariharan, 2021). Conversely, reducing expression of Rpk or ATPα using wing-specific RNAi reduces Hh signaling (Emmons-Bell and Hariharan, 2021). Direct modulation of V_mem_ via optogenetics regulates smoothened membrane localization, suggesting that bioelectricity regulates Hh signaling while also being regulated by it (Emmons-Bell and Hariharan, 2021). This suggests that Hh signaling and V_mem_ mutually reinforce each other (Figure 7).

Taken together, these studies suggest that all of the major canonical signaling pathways can be regulated by bioelectricity. Notch and Hh signaling regulate ion channel signaling while also being regulated by changes in bioelectricity. BMP and Wnt signaling both lie downstream of ion channel function and the activity of these pathways can be modulated by changes in bioelectricity.

## Direct Regulation of Transcription

Salvador Mafe, Michael Levin, and Javier Cervera have proposed that membrane potential could regulate transcription directly (Cervera et al., 2020; Levin, 2021). Thus, bioelectric signals could coordinate cellular outcomes between several cells within a developing tissue. In this model, bioelectric fields would control development on a tissue wide scale.

## Putting It All Together: Implications of Bioelectrical Signaling

It is becoming increasingly evident that ion channel function and the V_mem_ pattern in tissues is essential for development. Because ion channels play roles in nearly every essential developmental process including cell death, proliferation, cell polarity, migration, and regulation of the canonical developmental signaling pathways, this raises the exciting possibility that cells within tissues may use bioelectrical signaling as a high-level mechanism to coordinate the complex process of development of a tissue. While we described mechanisms on an individual cell level, in multicellular organisms, cells are working within a tissue. Thus, the contribution of ion channels to proliferation, apoptosis, cell migration, and signaling would impact the morphogenesis of a whole tissue or structure. For example, if the ion channels that impact proliferation and migration are inhibited during palatogenesis, the correct number of cells would not migrate to the palate shelves and would not proliferate adequately for palate shelves to reach one another and fuse at the midline, which would result in a cleft palate.

Calcium appears to be the major player in bioelectrical signaling. In each of the cellular processes which ion channels help regulate, calcium channels play the largest role of all of the ion channel types: calcium is the primary ion that acts in ion channel mediated regulation of cell death, working in both the ER related and mitochondria related cell death pathways, calcium acts at nearly every stage in the cell cycle to regulate proliferation, calcium channels are required for the establishment of cell polarity and for cell migration, and calcium plays a role in BMP, Notch, Wnt, and Hh signaling. ER regulating calcium channels are specifically required for many of these processes with SERCA, Stim, or Orai having been identified as necessary for nearly all of these cellular processes (Figure 2–Figure 6). Remarkably, these ER-calcium regulating channels are also necessary for the propagation of the calcium oscillations that occur spontaneously in developing tissues (Narciso et al., 2015; Ohno and Otaki, 2015; Restrepo and Basler, 2016; Balaji et al., 2017; Li et al., 2018), providing a potential link between calcium oscillations and these calcium-regulated cellular processes. The near universal existence of calcium oscillations in developing tissues paired with the known roles of calcium in multiple cellular processes and pathways raises intriguing possibilities for the role of calcium in guiding development. Calcium oscillations provide a mechanism by which cells could communicate in detailed ways. Oscillations contain many encoded variables—such as frequency, amplitude, and rate of change—that could each potentially carry information, allowing cells to fine-tune communication through subtle changes in oscillatory properties. Could it be that a variety of ion channels contribute to development by converging on regulation of intracellular calcium?

Cellular concentrations of one ion can influence concentrations of other ions. If the activity of one type of ion channel is impaired, concentrations the ion it conducts are altered, but concentrations of other ions can be changed as well. For example, membrane potential impacts cytoplasmic calcium levels. This is due to the high number of voltage-gated calcium channels that are able to respond to changes in V_mem_ by opening or closing and allowing or stopping calcium flux (Catterall, 2011). Many other channel types—including potassium, sodium, and chloride channels—are calcium sensitive and open upon calcium binding and lead to changes in V_mem_. Calcium-activated potassium channels in particular have been associated with calcium induced changes in V_mem_ (Lazzari-Dean et al., 2019). This feedback between calcium levels and V_mem_ allows both properties to mutually regulate each other.

The known roles of calcium oscillations and V_mem_ in development suggest a model in which these factors can be used for communication. A potential model can be imagined in which cells within growing tissues have varying V_mem_ values depending on their location within the tissue and the propagation of calcium oscillations. Because mechanical forces can induce changes in V_mem_ and calcium oscillations it is possible that the individual forces on each cell—which depend on the cell’s placement within a tissue—may help regulate this bioelectrical signaling. In turn, these bioelectrical signals could regulate the proliferation, death, cell polarity, and migration of each cell while also regulating the canonical developmental signaling pathways, ultimately guiding each cell to differentiate at the proper time and place to form the adult organism. Because bioelectrical signals such as calcium oscillations can encode multiple different variables, information could be fine-tuned to each cell. Whether a cell decides to divide, proliferate, die, or differentiate could depend not only on an overall level of a particular ion but on the combination of V_mem_ and calcium oscillation frequency or amplitude. Cells within tissues are interconnected via a vast network of gap junctions, so the bioelectrical state of each cell could in turn regulate the bioelectrical states of cells nearby. This model would suggest a mechanism by which cells are able to communicate rapidly and dynamically in response to perturbations such as damage during development.

## Next Steps in the Field of Bioelectricity

### Remaining Questions

Ion channels have emerged as important regulators of cell death, cell cycle regulation, cell polarity, migration, and canonical developmental signaling pathways. While the field of developmental bioelectricity is rapidly growing, there are still many questions to be answered.

It has only recently been discovered that ion channels play a role in well-established developmental signaling pathways such as BMP, Wnt, and Notch. How do the important molecular signaling pathways and ion channel signaling intersect? Ion channels clearly impinge upon BMP, hedgehog, notch, and wnt signaling pathways, but it is not well understood whether these effects are due to direct regulation of these pathways or due to downstream effects on cell cycle, cell death, or cell migration. While some research suggests that the role of ion channels in signaling is via a direct mechanism such as regulation of the secretion or trafficking of components of the BMP and notch signaling pathways (Dahal et al., 2017; Suisse and Treisman, 2019), more studies need to be done specifically investigating these potential mechanisms and whether these roles are conserved in development.

Another question in the field is whether the role of ion channel signaling in development is mediated primarily through impacting the transmembrane potential or through impacts on the spontaneous calcium oscillations that occur in developing organisms. As previously mentioned, both calcium oscillations and transmembrane potential are intrinsically related and influence each other. However, more work needs to be done to understand whether each property influences different aspects of development or whether both properties work within the same pathway. Much of the recent work in the field of developmental bioelectricity has focused on the patterns of V_mem_ across developing tissues and how these patterns might instruct development. However, while it is clear that transmembrane potential patterns across tissues are an important regulator of multiple cellular processes, there is evidence that the more slight but rapid changes in V_mem_ that occur via the propagation of calcium oscillations also play an important role in developing tissues. The discovery that many tissues spontaneously propagate calcium transients and waves, raises the possibility that cells could use ionic signaling to communicate across greater distances like neurons. There is evidence that ion channels may impact secretion dynamics of cell signaling pathways and that developing tissues propagate calcium waves. Are these calcium waves regulating morphogen secretion similar to the way action potentials regulate neurotransmitter release in neurons? Recent work in the *Drosophila* air sac primordium suggests that such a mechanism may be at work in cell types not traditionally thought of as excitable (Huang et al., 2019). In most tissue types, cells are connected via a vast network of gap junctions, enabling such changes in calcium to be used to communicate across tissues. More work needs to be done to investigate whether similar regulation of morphogens via calcium oscillations occurs in other cell and tissue types. With new tools in optogenetics and calcium sensing now readily available and improving, this research is now possible.

Another unanswered question in the field is how the transmembrane potential and calcium oscillations are regulated and coordinated on a tissue wide scale. If cells are using the transmembrane potential and calcium waves to communicate, then they must still be able to sense their position within a tissue to adopt the correct V_mem_. What upstream information allows cells to properly set their transmembrane potential and propagate calcium oscillations? Because many ion channels are sensitive to mechanical stresses, one hypothesis is that mechanical forces between cells within a tissue may guide ionic signaling. Mechanical forces play an important role in development, and mechanosensitive ion channels have been associated with cell processes such as development of cell polarity and migration (Mammoto and Ingber, 2010; Huang et al., 2015; Canales Coutino and Mayor, 2021). In fact, mechanical stressors have been found to induce calcium waves in many cell types and tissues (Godin et al., 2007; Tsutsumi et al., 2009; Narciso et al., 2015; Restrepo and Basler, 2016). Another possibility is that some of the well-established developmental signaling pathways may also shape calcium oscillations. The hedgehog signaling pathway is upstream of calcium waves, suggesting an alternative mechanism by which ion gradients may be regulated (Osawa et al., 2006; Belgacem and Borodinsky, 2011; Klatt Shaw et al., 2018; Li et al., 2018). More work needs to be done, however, to fully understand how calcium oscillations and V_mem_ patterns are established and regulated as tissues grow.

While there is a rising understanding of the role of ion channel function in each of the key developmental cellular processes of proliferation, cell death, migration, and molecular cell signaling, less is understood about how bioelectrical signaling may regulate these processes all together and how the bioelectrical patterns and each of these processes interconnect across a tissue. For example, oscillations in calcium play a role in progression of the cell cycle, induction of cell death, and activation of cell migration. How does the cell distinguish between these signals? When cytosolic calcium rises how does the cell know whether to divide, die, send a molecular signal, or migrate? One possibility is that the cell responds to narrow ranges of V_mem_ changes and calcium levels, with information potentially encoded within the frequency or amplitude of calcium oscillations. The resting membrane potential of a given cell in a tissue along with the active genes in each cell type, could dictate how easily calcium influx is induced and to what degree V_mem_ changes in response, which in turn could regulate whether the cell responds by dividing, dying, migrating, or propagating a developmental signaling pathway. For example, a specific cell that is already in a relatively depolarized region within a tissue would need less calcium influx to reach a specific calcium threshold than a cell in a relatively hyperpolarized region. However, calcium influx into the cell in the hyperpolarized region to reach the same final calcium threshold would result in a final amplitude with larger magnitude. Could these two different variables—time to threshold calcium level or final amplitude of calcium oscillation—encode different information? More work needs to be done on mapping the tissue wide patterns of bioelectrical signaling, the regions of calcium oscillation propagation, and developmental processes to broaden our understanding of how bioelectrical signaling may coordinate development at a tissue wide level.

### Potential Barriers

While much progress has been made in the field of developmental bioelectricity, there are still barriers that must be addressed. One difficulty is the many overlapping roles of ions, particularly calcium, in cell processes essential to development. Calcium acts as a messenger in a variety of developmental processes: regulating cell death, the cell cycle, cell polarity, migration and well-established developmental signaling pathways. Because many of these pathways impinge upon on the others attributing changes in developmental outcomes to specific pathways is difficult. More work needs to be done within controlled contexts such as cell culture systems to understand how calcium specifically impacts recognized signaling pathways apart from its impact on cell death and the cell cycle.

Another difficulty in elucidating the role of ion channels in development is that there is a limited ability to control a single ionic pathway without impacting others. Levels of calcium, potassium, sodium, and chloride all intersect with each other, which the levels of each ion impacting the levels of the others, making it difficult to distinguish roles of specific ion channels and ions. Much of the research in the field of developmental biology has focused on disruption or manipulation of specific individual genes to elucidate the roles of individual proteins. In the field of bioelectricity, it might be more beneficial to focus on overall changes in transmembrane potential and calcium oscillations rather than on specific channels as it is likely that these changes, rather than the specific ion channels themselves, are what regulate development.

## Concluding Remarks

Gaining a greater understanding of bioelectrical signaling will elucidate another complex pathway by which cells may coordinate development. This greater understanding of development is necessary to potentially open new avenues within medicine. Ion channel signaling is dependent on a network of interdependent ion channels and is not necessarily dependent on single individual types of channels. This means that pharmaceuticals that elicit changes in overall cell polarization rather than by acting on specific channels, could potentially regulate larger changes in morphogenesis which has not been previously possible. In regenerative medicine after trauma, for example, an understanding of bioelectric signaling may provide new avenues of directing tissue growth and healing. “Electroceuticals,” devices or drugs that induce bioelectrical changes in tissues, are already being investigated as potential mechanisms in medicine to improve healing (Levin et al., 2019).

In summary, ion channels play an essential role in development with bioelectricity regulating cell death, the cell cycle, proliferation, cell polarity, migration, and the canonical developmental signaling pathways. A greater understanding of the mechanisms by which ion channels act will reveal new avenues in medicine by which developmental disorders may be treated.
